# Literacy and Attitude Toward Suicide Among Doctors and Nurses: A Cross-Sectional Comparative Study

**DOI:** 10.7759/cureus.64032

**Published:** 2024-07-07

**Authors:** Roopika Jandial, Karthick Subramanian, Suriya Kumar, Eswaran Subramanian, Sivaprakash Balasundaram

**Affiliations:** 1 Department of Psychiatry, Government Medical College Udhampur, Udhampur, IND; 2 Department of Psychiatry, Mahatma Gandhi Medical College and Research Institute, Sri Balaji Vidyapeeth (Deemed to Be University), Puducherry, IND

**Keywords:** healthcare professionals, suicide literacy, suicidal behaviour, knowledge, awareness, attitudes

## Abstract

Introduction

Globally, suicide remains a major public health problem and is the second most common cause of death in the 15-45 years age group. Awareness about suicide and the attitude of healthcare professionals regarding suicide are crucial factors determining the care for suicidal behaviors. The present study aimed to compare suicide literacy and attitude toward suicide between doctors and nurses and to explore the association between suicide literacy and attitude toward suicide.

Methods

A cross-sectional comparison study was conducted between doctors and nurses in a tertiary healthcare center in southern India. The demographic characteristics and work and suicide care experience were recorded using a semi-structured proforma. Literacy of Suicide Scale - Short Form (LOSS-SF) and Attitude Toward Suicide Questionnaire (ATTS) were used to assess the awareness about suicidal behaviors and attitudes toward suicide, respectively. Appropriate statistical tests were used to compare the outcome measures between doctors and nurses with the statistical significance set at p ≤ 0.05.

Results

A total of 600 healthcare professionals (doctors (n=300) and nurses (n=300)) were included. The doctors were older, had longer work experience, and demonstrated better suicide literacy, yet harbored more negative attitudes toward suicide than nurses. Among doctors, increasing levels of suicide literacy were negatively correlated with positive attitudes toward suicide. Among nurses, increasing age was negatively correlated with healthy attitudes toward suicide.

Conclusion

Doctors had better awareness of suicidal behaviors yet displayed negative attitudes toward people with suicidal behaviors. Despite having less suicide awareness, nurses exhibited positive attitudes toward suicide. Suicide literacy and attitudes toward suicide can have complex relationships mediated by multiple factors.

## Introduction

Suicide remains a major global public health concern with profound impacts on individuals, their families, and society at large. Annually, more than 800,000 people succumb to suicide, along with an even higher number of suicide attempts [1]. Treatment-seeking behaviors among suicidal patients depend on various factors: individual (stigma, self-management of stress), social (lack of support from family and friends), and healthcare system-related (limitation of resources, lack of knowledge, and unfavorable attitudes) reasons [2,3]. 

Among healthcare system-related factors, the awareness of suicidal behaviors and the attitudes displayed by healthcare professionals toward the persons recovering from suicide attempts are crucial factors in the treatment and care provided to suicidal patients [4,5]. Such factors play significant roles early in the suicidal process while screening for the risk of impending suicide as most attempters often approach their family physicians or general practitioners for help within a short interval before the actual attempt [6-9].

Suicide literacy is defined as the “awareness of the causes, risk factors, signs or symptoms, and treatments for suicidal behaviors,” which plays a crucial role in identifying people with suicide risk [10]. Adequate awareness of suicidal behaviors among healthcare professionals is crucial for early identification of high-risk groups, effective therapeutic communication, and comprehensive care planning for suicidal patients [11]. In addition, suicide literacy among healthcare professionals strongly influences their attitudes when providing care to patients with suicidal behaviors [12]. Only recently, a systematic review of global studies on healthcare providers' (HCPs’) knowledge and attitudes analyzed the interplay between suicide-related awareness and attitudes among HCPs. It revealed that better levels of suicide-related awareness were associated with more positive attitudes toward suicidal behaviors [13].

Apart from awareness about suicide, the attitudes of health care professionals (doctors and nurses) displayed during the encounters with people admitted for suicidal behaviors influence their skills in assessing and managing suicide risk [14,15]. Attitudes toward suicide can be defined as “the set of views or cognitions about suicide behavior among the population” [16]. An earlier review highlighted that hospital staff often harbored negative attitudes toward patients with self-harm behaviors [17]. While some studies showed that doctors exhibited negative attitudes toward suicide-related behaviors [17,18], contrasting studies revealed that doctors did display positive attitudes toward suicide and endorsed the view that suicide is preventable [19]. Similarly, the findings are mixed regarding the attitudes of nurses toward suicide. While some studies have found that nurses exert positive attitudes toward suicide [20-22], contrasting studies showed that nurses could have negative attitudes toward patients with suicidal behaviors [23-26].

In developing nations like India, where the annual suicide rates are incremental, assessing the factors that influence the care of people with suicidal behavior becomes important. Recent Indian literature sheds light on the attitudes toward suicide exhibited by a variety of healthcare professionals such as more focus on physical issues rather than psychological support [27], adverse attitudes toward suicide attempters among junior resident doctors [28], unfavorable attitudes in medical undergraduates [29], reduced suicide literacy and awareness among nursing students [14], and beneficent attitudes toward suicide among nursing teachers [30], Nevertheless, awareness-promoting measures such as focused workshops, brief lectures, and comprehensive educational programs have been shown to promote suicide literacy and attitudes toward suicide among medical undergraduates and nurses [31-33].

Though suicide-related awareness and attitudes toward suicide have been studied, the interplay between suicide literacy and attitudes toward suicide and profession-related disparities in suicide-related cognitions have not been adequately studied in India. Such explorations can provide deeper insights about how awareness and attitudes of healthcare professionals influence each other while dealing with suicidal patients, and can reiterate the need for dedicated suicide-related awareness activities among doctors and nurses. Hence, the current study aimed to explore the prevailing awareness of suicide and attitudes toward suicide behaviors among healthcare professionals (doctors and nurses). The objectives of the study were to assess and compare the patterns of suicide literacy and attitudes regarding suicidal behavior between doctors and nurses, and to explore the factors associated with suicide literacy and attitudes.

## Materials and methods

Study setting, design, and participants

The present cross-sectional, observational study was done at Mahatma Gandhi Medical College and Research Institute, Sri Balaji Vidyapeeth, Puducherry, India, between March 2019 to July 2020. Non-probabilistic convenience sampling was considered for the study due to the exploratory nature of the research and time constraints in reaching out to healthcare professionals during busy clinic hours. The sample size was calculated based on previous similar studies [14,29]. The sample universe consisted of healthcare professionals, including postgraduate students (n=185), faculty doctors (n=160), and nurses (n=410). Individual healthcare professionals were approached in two methods: in-person visits in medical wards and outpatient departments and through email correspondence with an invitational email comprising the participant information sheet and informed consent form. Upon consenting to participation, the healthcare professionals were included in the study. Non-consenting participants were excluded from the study. The response rates were 81.1%, 93.7%, and 73.1% among the postgraduate students, doctors, and nurses, respectively.

Instruments and tools

Semi-structured Proforma

A semi-structured proforma was used to collect the demographic and exposure characteristics of the participants. The clinical exposure of each healthcare professional or student was assessed by enquiring duration spent or having been posted in casualty (casualty experience), post-operative settings (post-operative experience), intensive care unit (ICU) wards (ICU experience), and the duration spent in suicide care (time spent in consultation for or care tended toward suicidal patients) (suicide care experience). The family history of suicide was explored in the subjects through three questions (developed by the study investigator based on “contact with suicide problem” section of the Attitudes Toward Suicide Questionnaire (ATTS) scale): “Do any of your close relatives expressed suicidal ideation or attempted suicide?” (past suicidal behavior), “Do any of your close relatives currently have suicidal thoughts at present?” (current suicidal thoughts), “Do any of your close relatives have committed suicide?” (completed suicide). All three questions had three options namely “yes,” “no,” and “do not want to answer,” respectively.

Literacy of Suicide Scale-Short Form (LOSS-SF)

LOSS-SF* *[34], developed by Calear et al. (2022) consisted of 12 questions related to signs and symptoms and risk factors of suicide. The questions were rated with yes/no answers. The scale has four literacy themes: a) risk factors, b) signs/symptoms, c) cause/nature, and d) treatment/prevention for the identification of strengths and weaknesses in a response. Increasing scores indicate better awareness of these factors. The scale has demonstrated good reliability, excellent validity, and a high internal consistency (Cronbach’s α=0.83) [35].

ATTS

ATTS [36] consisted of three sections: "contact with suicide problem," "attitudes," and "questions about self." The attitudes component was used as an ATTS questionnaire, which had 40 items (item numbers 4 to 43 in the ATTS scale). Items 41 and 42 were dropped as they explored impending suicidal thoughts in apparently healthy volunteers. The rest of the items were used to analyze the scores of 10 domains in ATTS: suicide as a right: endorses opinion about suicide as a right of an individual; incomprehensibility: endorses an idea about not understanding the reason behind suicide; non-communication: endorses opinion about people who threaten to be suicidal will not commit suicide; preventability: endorses opinion that all suicides are not preventable; tabooing: endorses idea that enquiring about suicide thoughts can evoke fresh suicidal thoughts; normal/common: endorses opinion that everyone has considered suicide at some point in their lives; suicidal process: endorses idea that suicide can usually be considered for a long time; relation-caused: endorses opinion that suicides are often due to interpersonal conflicts and loneliness; preparedness to prevent: endorses idea that it is human duty to prevent suicide; resignation: endorses idea that some people may consider suicide to be the only solution for their situation. Higher scores in each domain indicate a greater degree of endorsement of that domain on the ATTS scale. The scale has demonstrated fair reliability and validity with moderate internal consistency (Cronbach’s α=0.60) [35].

Study procedure

After providing study-related information and obtaining written informed consent, the participants were provided three forms (physical or digital through Google® forms): sociodemographic proforma, LOSS, and ATTS scales. Permissions were obtained from the developers of LOSS and ATTS for use in the study. All three forms were self-rated by the study participants. The requisite details about the sociodemographic profile, occupation, and clinical exposure to suicide care were recorded in a semi-structured proforma. Upon completion, the participants recorded their responses to the LOSS and ATTS scales. Since the participants could comprehend English, the original, unmodified scales were provided for data collection. Both LOSS-SF and ATTS have been validated and previously used in studies involving Indian healthcare professionals [37,38]. Confidentiality of the information was ensured by assigning an identification number to the responses mentioning the group identity alone (e.g., FAC001 for faculty, PG001 for postgraduate (PG), NUR001 for nurses, and so on).

After data collection, the participants were divided into two groups namely doctors (including faculty and PGs) and nurses. Data comparison was done based on sociodemographic data (age, gender, religion, area of domicile, marital status, and type of family), suicidal care exposure variables - casualty experience, post-operative ward experience, ICU experience, and suicide care in a lifetime (in months), number of suicidal patients cared for in a lifetime, and LOSS score - the percentage of correct answers given and ATTS scores.

The study protocol was approved by the Institutional Human Ethics Committee of Mahatma Gandhi Medical College and Research Institute, Puducherry (MGMCRI/PGD/02/2019/06) and conformed to the Helsinki Declaration of medical research ethical principles. 

Statistical analyses

The distribution of continuous variables was depicted using means and standard deviation, and that of categorical variables using frequency and percentages. The study sample was divided into two groups, namely doctors (with faculty and PGs) and nurses, and inferential analyses were performed. Subgroup analyses were performed in both groups based on age, group, gender, religion, area of domicile, family type, and number of suicidal patients cared for during their professional tenure. Comparison of means of normally distributed continuous variables was done using independent sample t-test and one-way ANOVA. Kruskal Wallis and Mann-Whitney U tests were used to compare means of non-normal distributions. The differences between categorical variables were computed using the chi-square test. Correlation analyses were done among the variables age, LOSS score, and domain scores of the ATTS scale. Data analysis was performed using SPSS Statistics for Windows, Version 17 (Released 2008; SPSS Inc., Chicago, United States). Statistical significance was set at p ≤ 0.05.

## Results

Comparison of sociodemographic profile between doctors and nurses

A total of 300 doctors (150 faculty and 150 postgraduate students) and 300 nurses participated in the study. While most participants belonged to the Hindu religion, the doctors were significantly older, hailed from an urban domicile, and lived in nuclear families when compared to nurses. A greater proportion of nurses were female and married. Both groups had a greater representation of females (Table 1).

**Table 1:** Comparison of socio-demographic variables among doctors and nurses *p<0.05, statistically significant

Variable	Doctors (n=300) mean (SD) or frequency (%)	Nurses (n=300) mean (SD) or frequency (%)	Test characteristic	p-value
Age	32.54 (9.01)	26.90 (3.28)	t=10.178	p<0.001*
Male	147 (49.0%)	47 (15.7%)	χ^2^=77.734	p<0.001*
Female	152 (50.7%)	253 (84.3%)		
Transgender	1 (0.3%)	0		
Hindu	243 (81.0%)	230 (76.7%)	χ^2^=11.968	p=0.018*
Christian	25 (8.3%)	38 (12.7%)		
Muslim	22 (7.3%)	31 (10.3%)		
Others	1 (0.3%)	0		
Atheist	9 (3.1%)	1 (0.3%)		
Unmarried	149 (49.7%)	110 (36.7%)	χ^2^=16.123	p=0.003*
Married	147 (49.0%)	189 (63.0%)		
Widowed	0	1 (0.7%)		
Divorced	1 (0.3%)	0		
Separated	3 (1.0%)	0		
Urban	256 (85.3%)	89 (29.7%)	χ^2^=190.206	p<0.001*
Rural	44 (14.7%)	211 (70.3%)		
Nuclear	215 (71.7%)	100 (33.3%)	χ^2^=92.784	p<0.001*
Joint	71 (23.7%)	185 (61.7%)		
Three generation	14 (4.7%)	15 (5.0%)		

Comparison of personal experience and clinical experience with suicide care between doctors and nurses

When compared to doctors, nurses reported higher rates of family history of current suicidal thoughts and past completed suicides (Table 2).

**Table 2:** Comparison of personal exposure variables between doctors and nurses *p<0.05, statistically significant; **items taken from Attitude Toward Suicide Questionnaire

Variable		Doctors (n=300) n (%) or mean rank	Nurses (n=300) n (%) or mean rank	Test statistic	p-value
Personal exposure variables**					
Close relative expressed suicidal ideation or attempted suicide	Yes	86	83	χ^2^=1.184	0.553
	No	195	191		
	Don’t want to answer	19	26		
Close relative currently has suicidal thoughts	Yes	21	44	χ^2^=11.083	0.004*
	No	268	239		
	Don’t want to answer	11	17		
Close relative has committed suicide	Yes	48	75	χ^2^=8.246	0.016*
	No	239	209		
	Don’t want to answer	13	16		

It was found that doctors had a greater duration of experience in casualty and post-operative wards than nurses. The doctors also recorded a longer duration of suicide care experience than nurses (Table 3).

**Table 3:** Comparison of clinical exposure variables between doctors and nurses *p<0.05, statistically significant; **items taken from Attitude Toward Suicide Questionnaire; ICU: intensive care unit

Variable	Doctors (n=300) n (%) or mean rank	Nurses (n=300) n (%) or mean rank	Test statistic	p-value
Clinical exposure variables				
Casualty experience (in months)	315.04	285.96	U=40637.500	0.034*
Post-operative experience (in months)	315.06	285.94	U=40631.500	0.034*
ICU experience (in months)	294.93	306.07	U=43329.000	0.419
Suicide care experience (in months)	367.58	233.42	U=24877.000	<0.001*

Comparison of literacy of suicide and attitudes toward suicide between doctors and nurses

The literacy of suicide was estimated as the average correct score, and the same was compared between the two groups (Table 4). The mean correct score in LOSS was higher in doctors than in nurses.

**Table 4:** Comparison of percent correct score of Literacy of Suicide Scale (LOSS) between doctors and nurses *computed as (total no. of correct responses by participant/12)*100; **statistical significance set at p<0.05

Variable	Group	Mean (SD)	t-value	p-value
Mean correct score of LOSS*	Doctors (n=300)	70.58 (15.336)	13.292	0.036**
	Nurses (n=300)	54.81 (13.69)		

The attitudes toward suicide domain scores were compared among the sample by comparing 10 domains of attitude toward suicide scale. It was found that the doctors had a greater endorsement of ideas than the nurses in domains such as suicide is an individual’s right, suicide threat will not end in an attempt, suicides are unpreventable, suicidal ideas are incited by enquiring about suicide, suicidal ideas are prevalent, suicides are often due to interpersonal conflict and loneliness, and suicide can be considered as the only solution for problems among the general population. In contrast, the nurses reported a more significant endorsement that it is a human duty to prevent suicide (Table 5).

**Table 5:** Comparison of ATTS domain scores between doctors and nurses *p<0.05, statistically significant; ATTS: Attitudes Toward Suicide Scale

Variable	Doctors (n=300) mean (SD)	Nurses (n=300) mean (SD)	t-value	p-value
1. Suicide right	20.53 (4.91)	18.61 (4.38)	5.038	<0.001*
2. Incomprehensibility	13 (3.48)	12.50 (3.08)	1.865	0.063
3. Non-communication	14.01 (3.6)	12.94 (3.18)	3.864	<0.001*
4. Preventability	8.86 (1.96)	9.61 (2.40)	-4.151	<0.001*
5. Tabooing	11.51 (2.28)	8.25 (2.14)	18.040	<0.001*
6. Normal/common	8.75 (2.41)	7.61 (2.33)	5.890	<0.001*
7. Suicidal process	6.75 (1.42)	6.81 (1.61)	-.513	0.608
8. Relation-caused	12.16 (2.83)	10.63 (2.91)	6.557	<0.001*
9. Preparedness to prevent	5.39 (1.92)	6.93 (1.92)	-10.157	<0.001*
10. Resignation	7.54 (1.89)	6.03 (2.03)	9.475	<0.001*

Inter-relationship between age, suicide literacy, and attitudes

Correlation analysis between age and literacy of suicide score was done among doctors and nurses. However, there was no statistically significant difference in both groups. Increasing age among nurses was associated with greater endorsement of negative attitudes such as “all suicides are not preventable” and “enquiring about suicide can incite suicidal thoughts.” Similar associations were not observed among doctors (Table 6).

**Table 6:** Correlation between age and attitudes toward suicide - doctors and nurses * p<0.05, statistically significant; ATTS: Attitudes Toward Suicide Scale

ATTS domain	Age	
	Doctors (Pearson’s r, p-value)	Nurses (Pearson’s r, p-value)
1. Suicide right	0.035 (0.544)	0.065 (0.260)
2. Incomprehensibility	-0.110 (0.061)	0.068 (0.239)
3. Non-communication	0.018 (0.750)	0.019 (0.742)
4. Preventability	-0.084 (0.148)	0.123 (0.034*)
5. Tabooing	0.094 (0.104)	0.139 (0.016*)
6. Normal/common	0.025 (0.661)	0.053 (0.358)
7. Suicidal process	-0.005 (0.927)	-0.007 (0.908)
8. Relation-caused	-0.018 (0.763)	-0.005 (0.933)
9. Preparedness to prevent	0.029 (0.611)	0.101 (0.082)
10. Resignation	-0.057 (0.329)	0.046 (0.426)

A correlation between literacy of suicide score and attitudes toward suicide was done among doctors and nurses, which revealed that increased awareness about suicidal behaviors was associated with increased chances of endorsement that “reasons for suicide are incomprehensible, suicide threat will not end in an attempt, suicidal ideas are incited by enquiring about suicide, and suicides are often due to interpersonal conflict and loneliness.” It was also noted that increased suicide literacy is associated with reduced chances of endorsing that “suicidal ideas are very common, and prevention of suicide is a human commitment.” Among nurses, increased suicide literacy is associated with increased chances of endorsing that suicide can usually be considered for a long time, and suicides are often due to interpersonal conflict and loneliness (Table 7).

**Table 7:** Correlation between literacy of suicide score and attitudes toward suicide - doctors and nurses *p<0.05, statistically significant; LOSS: Literacy of Suicide Scale; ATTS: Attitudes Toward Suicide Scale

ATTS domain	Percent correct response in LOSS	
	Doctors (Pearson’s r, p-value)	Nurses (Pearson’s r, p-value)
1. Suicide Right	0.085 (0.143)	0.136 (0.081)
2. Incomprehensibility	0.132 (0.022*)	0.108 (0.061)
3. Non-communication	0.263 (<0.001*)	0.080 (0.165)
4. Preventability	0.047 (0.413)	-0.041 (0.483)
5. Tabooing	0.303 (<0.001*)	0.095 (0.101)
6. Normal/common	-0.122 (0.034*)	0.011 (0.844)
7. Suicidal process	-0.107 (0.064)	0.153 (0.008*)
8. Relation-caused	0.136 (0.019*)	0.139 (0.016*)
9. Preparedness to prevent	-0.180 (0.002*)	0.042 (0.469)
10. Resignation	0.084 (0.146)	0.095 (0.102)

Subgroup analyses

Subgroup analyses were performed in both the doctors’ and nurses’ groups based on age group (21-30, 31-40, 41 and above), gender, religion, area of domicile, family type, and number of suicidal patients cared for during their professional tenure. However, the results were inconclusive.

## Discussion

The present cross-sectional study aimed to assess the prevailing awareness about suicide (suicide literacy) and attitudes toward suicide among healthcare professionals of a tertiary care general hospital in southern India. A total of 600 HCPs (300 doctors (150 faculty and 150 postgraduates) and 300 nurses) who worked in various departments participated in the study. Suicide literacy and attitudes toward suicide were assessed using standard scales similar to previous studies [29,37-40].

Sociodemographic characteristics of healthcare professionals

The majority of the HCPs belonged to the Hindu religion, similar to that reported in previous Indian studies [29,41]. The difference between doctors and nurses based on family type and domicile could be due to site-specific demographic reasons.

Suicide literacy and attitudes: doctors versus nurses

The observation of better levels of suicide literacy among doctors than the nurses in our sample is similar to previous studies where nurses demonstrated lower suicide literacy levels when compared to doctors [12,41,42]. The difference could be explained by doctors being older and having longer experience in handling patients with suicidal behaviors than nurses. The difference could also be due to longer and more intensive training among physicians in suicide care than the nurses as reported by previous studies [43].

However, though doctors had a longer professional experience and had reported better awareness of suicidal behaviors, a greater number of doctors harbored negative attitudes toward suicidal behaviors in considering “suicide as one’s right, as a justified solution for one’s problems, unpreventable, easily inducible by inquiry, and not all threats end in an attempt.” The findings are similar to those reported in previous studies, which revealed that compared to nurses, doctors displayed more negative rather than positive attitudes [17,43,44]. Some authors have posited that doctors’ negative attitudes toward suicide could be due to less empathy displayed during patient encounters [45]. Socio-cultural factors like a religious condemnation of suicide [46,47], internalization followed by a reflection of social stigma on suicide by doctors [48], and viewing suicide as a punishable offense could possibly explain the negative attitudes toward suicide [49].

Despite the short duration of experience and low levels of suicide literacy, nurses endorsed positive attitudes such as committing oneself to prevent suicide. Earlier studies among nurses revealed similar findings that nurses believed that prevention of suicide is their responsibility and that every act of suicide could be prevented [17,30,50]. This could be explained by the sample consisting of predominantly female nurses who tend to be more sympathetic toward suicide behaviors than their male counterparts, a greater burden of family history of suicide attempts/deaths among nurses’ relatives than those of doctors, leading to a priming of the nurses’ mindset to harbor favorable attitudes toward suicide attempters [13,30,51,52]. Studies reveal that a combination of training, direct patient interaction, holistic care approaches, and cultural factors within the nursing profession contribute to more positive attitudes toward suicide compared to doctors [53]. Though previous studies reveal that with increasing age, nurses displayed better attitudes toward suicide, our study found that with increasing age, nurses tend to express more negative attitudes toward suicide [54,55]. This could be due to the complex interplay of professional experience, burnout, compassion fatigue, and cultural factors in shaping attitudes toward suicide among older nurses [24,56]. Earlier studies have shown that diminished empathy and professional burnout were linked to greater cynicism and negative attitudes toward people with suicidal behavior [57,58]. The findings, however, need further evaluation of the psychological and psychosocial factors associated with unfavorable attitudes toward suicide among healthcare professionals.

Strengths and limitations

The present study adds to the scarce literature comparing suicide awareness and attitudes between doctors and nurses in Indian settings by exploring the inter-relationship between suicide literacy and attitudes toward suicide among doctors and nurses, and the association between their clinical exposure characteristics and suicide-related attitudes [14,27-29,41].

The limitations of the study are that we did not employ standard formulae to calculate sample size which might reflect reduced statistical power and limit the generalizability of the study’s findings. There is a possibility of selection bias due to the use of purposive sampling in the study. The present study did not include comparisons based on years of experience (early-career healthcare professionals versus mid- or late-career healthcare professionals), and comparisons across various specialties/departments where the healthcare professional or student was employed or studying. This was avoided due to the non-probabilistic sampling limitation already minimizing generalizability, and the sample size was calculated based on a primary comparison of interest (doctors versus nurses).

Future interdisciplinary studies are needed to explore the level of awareness and attitudes toward suicide among other HCPs such as psychologists and social workers, and gatekeepers such as educators, first responders, community leaders, etc. Apart from quantitative studies, qualitative research in this domain may shed new insights into the awareness and attitudes toward suicide among HCPs. Similar studies should be undertaken among crucial suicide gatekeepers of society such as the teachers, family physicians, and primary health care workers who might harbor different levels of knowledge and awareness related to suicidal behaviors.

## Conclusions

This present study revealed that doctors had higher suicide literacy than nurses and yet displayed more negative attitudes toward people with suicide-related behavior. However, despite having a lesser degree of suicide literacy, nurses expressed a more positive attitude toward people with suicide-related behaviors. Dedicated suicide awareness initiatives in the workplace and organizing exclusive continued education programs focusing on bedside suicide risk assessment for doctors and nurses will have desirable effects on literacy and attitudes toward suicide among healthcare professionals.
